# Evaluation for endotoxin in intraocular materials used during phacoemulsification surgery using a recombinant factor C assay

**DOI:** 10.1111/vop.13149

**Published:** 2023-09-22

**Authors:** Taylor A. Opgenorth, Eryn L. Opgenorth, J. Seth Eaton, Ellison Bentley

**Affiliations:** ^1^ Department of Surgical Sciences, School of Veterinary Medicine University of Wisconsin Madison Wisconsin USA; ^2^ Virology Section Wisconsin Veterinary Diagnostic Laboratory Madison Wisconsin USA

**Keywords:** cataract, endotoxin, fibrin web, phacoemulsification, recombinant factor C, toxic anterior segment syndrome

## Abstract

**Objective:**

Cataract surgery remains the sole method to resolve blindness secondary to cataract formation. One complication includes fibrin web formation post‐operatively. This study aimed to investigate the presence of endotoxin within materials used during cataract surgery as a possible cause of fibrin web phenomenon.

**Methods:**

Preservative‐free epinephrine, heparin, viscoelastic devices, and intraocular lenses were collected for evaluation. Various manufacturers and manufacturing lot numbers were used when available. Viscosity of viscoelastics was reduced by incubating samples with human recombinant hyaluronidase. Intraocular product (IOL) packaging fluid was collected and stored for testing. The IOLs were then washed with a sterile balanced salt solution, incubated at 37°C for 48 h, and then fluid was collected for testing to mimic intraocular placement. Samples were tested using a commercially available rFC kit. Fluorescence was measured at time zero and after 1 h using a fluorescence microplate reader. The change in fluorescence was corrected for blank fluorescence and plotted to a standard curve.

**Results:**

Endotoxin levels were below the limit of detection (0.05 EU/mL) in all samples. Incubation of IOLs at intraocular temperature did not increase extraction of endotoxin.

**Conclusion:**

Endotoxin was not identified in any tested sample, including those used in cases of fibrin web formation post‐phacoemulsification. As fibrin webs are often observed episodically, it is possible that endotoxin levels may vary between batches, or that endotoxin is not related to fibrin formation.

## 1 INTRODUCTION

Cataract surgery remains the sole method to resolve blindness secondary to cataract formation. One complication that remains elusive is the development of fibrinous uveitis, characterized by fibrin accumulation within the anterior chamber, lens capsule, and around the intraocular lens, typically within 1 week of surgery.^1^ The primary contributing factors to fibrin formation are unknown. A recent study found an increased risk of formation with increased patient age, longer surgery time, and particular combinations of artificial lens and viscoelastic types.^2^ Cases of fibrin web formation are often observed in clusters, leading to the concern of an external source or product being the inciting factor.

The fibrin web phenomenon shares some similarities with toxic anterior segment syndrome (TASS) in humans.^1^, ^2^ TASS is defined as sterile post‐operative anterior uveitis, characterized by marked corneal edema and hypopyon with limited anterior cellular reaction.^3^ Onset is typically acute, within days, but late‐onset cases have been described.^4^ TASS has been associated with inadequate cleaning of surgical instruments, detergent residues on instruments, intraocular product (IOL, viscosurgical device, and trypan blue) contamination with endotoxin or manufacturing residues, solution pH, presence of preservatives, and intracameral drug reactions.^3^, ^4^ One outbreak revealed endotoxin concentrations of up to 0.9 EU/mL in contaminated BSS samples, while others identified endotoxin concentrations of >0.5 EU/mL in specific ophthalmic viscosurgical devices.^3^ In veterinary medicine in 2015, high concentrations of endotoxin (0.7–2.0 EU/mL) in water sources used during instrument cleaning were linked to similar incidents.^5^ Recent work evaluating the microbiota within ophthalmic surgical materials discovered endotoxin within viscoelastic samples.^2^ The authors identified significant differences in concentration between manufacturers as well as between lots of individual manufacturers.

Endotoxin, or lipopolysaccharide (LPS) are molecules found within the outer membrane of gram negative bacteria.^6^, ^7^ Endotoxins bind various toll‐like receptors (TLRs), which induce the release of pyrogenic cytokines, thus initiating the inflammatory cascade.^6^ Since 1983, testing for endotoxin in the biomedical industry has relied on the horseshoe crab, *Limulus polyphemus*, which when infected by intravascular endotoxin can develop fatal coagulation.^6^ The assay requires collection of hemolymph from horseshoe crabs; approximately 600 000 are wild‐caught yearly for collection and approximately 30% die from the procedure.^8^ Hemolymph contains a protein, Factor C, which when bound by endotoxin initiates the coagulation cascade. Limulus amebocyte lysate (LAL) assays are inherently prone to batch variability due to age, sex, and health of the harvested animals. Various proteins present in hemolymph can result in both assay inhibition and enhancement as well as induce the cascade in response to non‐endotoxin pyrogens.^6^ A sustainable and highly predictable assay using recombinant Factor C (rFC) that does not rely on the horseshoe crab has been recently developed and validated.^7^

This study aimed to investigate endotoxin concentration within materials used during cataract surgery as a possible cause of the fibrin web phenomenon using the rFC assay. Products that were evaluated include ophthalmic viscosurgical devices (OVD), intraocular lens (IOL) packaging fluid, heparin, and epinephrine.

## 2 METHODS

### 2.1 Ophthalmic viscoelastic device preparation

Ophthalmic viscoelastic devices (OVDs) from multiple manufacturers were obtained, with multiple lot numbers used when available (Table 1). One‐half milliliter of viscoelastic was transferred to a pyrogen‐free vial and combined with 30 U (0.2 mL) of human recombinant hyaluronidase (Halozyme Therapeutics, Inc.). This method was used to reduce sample viscosity and improve endotoxin detection per previous study guidelines.^9^ The OVD/hyaluronidase combination was vortexed at high speed for 10 s, then shaken at room temperature on a platform shaker at 55 rmp for 24 h. Samples were immediately transferred to a sterile black‐walled 96‐well microplate for testing.

**TABLE 1:** Specific materials tested for endotoxin including OVDs (viscoelastics), IOLs (intraocular lenses), and phacoemulsification fluid additives.

Material	Company	Company location	Product model	Endotoxin concentration (EU/mL)
OVD	an‐vision	Utah, USA	an‐bfh 1.8% lot′	<0.05
OVD	an‐vision	Utah, USA	an‐bfh 1.8% lot″	<0.05
OVD	Bausch + Lomb	New Jersey, USA	Amvisc® Plus 1.6% lot′	<0.05
OVD	Bausch + Lomb	New Jersey, USA	Amvisc® Plus 1.6% lot″	<0.05
OVD	CARAlife	California, USA	CaraVisc 1.8%	<0.05
OVD	Kite Vision Vet	California, USA	KiteVisc™ 1.8%	<0.05
OVD	Kite Vision Vet	California, USA	KiteVisc™ 3%	<0.05
OVD	I‐MED Animal Health	Quebec, Canada	i‐Visc® 1.8% lot′	<0.05
OVD	I‐MED Animal Health	Quebec, Canada	i‐Visc® 1.8% lot″	<0.05
IOL	an‐vision	Utah, USA	MD6‐14	<0.05
IOL	an‐vision	Utah, USA	MD6‐12 lot′	<0.05
IOL	an‐vision	Utah, USA	MD6‐12 lot″	<0.05
IOL	Bausch + Lomb	New Jersey, USA	Acrivet 60V‐14	<0.05
IOL	Bausch + Lomb	New Jersey, USA	Acrivet 60V‐13	<0.05
IOL	Bausch + Lomb	New Jersey, USA	Acrivet 60V‐12	<0.05
IOL	CARAlife	California, USA	CLV‐14	<0.05
IOL	CARAlife	California, USA	CLV‐13	<0.05
IOL	CARAlife	California, USA	CLV‐12	<0.05
IOL	VisTrill® Vet	Ontario, Canada	GVF7014 (feline model)	<0.05
IOL	Kite Vision Vet	California, USA	CLV‐13	<0.05
Epinephrine	BPI Labs LLC	Wyoming, USA	Preservative Free 1 mg/mL	<0.05
Epinephrine	BPI Labs LLC	Wyoming, USA	Preservative Free 1 mg/mL	<0.05
Heparin	Fresenius Kabi	Bad Homburg, Germany	Preservative Free 1000 U/mL	<0.05

*Note*: The ′ and ″ denote different lot numbers when multiple of the same product model were available.

Abbreviations: IOL, intraocular product; OVD, ophthalmic viscosurgical devices.

### 2.2 Intraocular lens preparation

Canine (with the exception of one feline) intraocular lenses, from various lots when possible, were acquired from various manufacturers (Table 1). Packaging fluid was transferred to endotoxin‐free vials for storage at −20°C. The intraocular lenses were washed with 35 mL of sterile endotoxin‐free balanced salt solution (BSS) using an 18 g needle attached to a syringe, mimicking intraoperative washing of IOLs prior to intraocular injection. IOLs were not handled directly; only packaging was manipulated. The IOL packaging was closed and the IOLs were incubated in BSS for 48 h at 37°C. After incubation, fluid was removed and transferred to an endotoxin‐free vial for storage at −20°C until testing.

### 2.3 Miscellaneous intraocular fluid preparation

Preservative‐free heparin (Fresenius Kabi) and epinephrine (BPI Labs LLC), with multiple lot numbers when possible, were tested. When necessary, pH was adjusted to fall into the acceptable range for the assay (between 6 and 8) using endotoxin‐free sodium hydroxide and hydrochloric acid.

### 2.4 Recombinant factor C assay

All instruments used (pipette tips, vials, plates) were endotoxin/pyrogen‐free per manufacturer labeling. The rFC assay was performed per manufacturer guidelines (Lonza PyroGene™ Recombinant Factor C Endotoxin Detection Assay; Lonza Walkersville, Inc.). Negative and positive controls were included in each run. Fluorogenic substrate, buffer, and enzyme solution were added to all wells. Samples were run in duplicates. Fluorescence was measured with a microplate reader using 380/440 nm excitation/emission wavelengths at time 0 and 1 h later. A set of known concentrations of bacterial endotoxin were used to develop a standard curve. The correlation coefficient (*R* ^2^ value) for the standard curves was between .990 and .999. The standard curve was used to find endotoxin concentration within samples. The Lonza PyroGene™ Recombinant Factor C Endotoxin assay is labeled to detect endotoxin concentrations between 0.005 and 5 EU/mL; however, instrument constraints only allowed a minimum detection of 0.05 EU/mL.

## 3 RESULTS

This study confirmed a relatively simple and efficient method to detect endotoxin within intraocular surgery materials that do not rely on the collection of horseshoe crab hemolymph. Spiked samples and positive controls were consistently detected above 0.05 EU/mL. All OVD, IOL samples (before and after incubation), heparin, epinephrine, and negative control endotoxin concentrations were below the limit of detection (0.05 EU/mL).

## 4 DISCUSSION

This study did not identify endotoxin concentrations above 0.05 EU/mL in any tested material, which is significantly lower than allowable endotoxin concentrations per FDA guidelines (<0.2 EU/mL or per device).* However, FDA regulations for humans may not translate accurately to animal use. Evaluation of response to intracameral endotoxin in rabbits revealed a dose‐dependent accumulation of anterior chamber cells and fibrin at 6 h after injection in all groups, including the lowest 0.02 EU/eye group.^10^ More inflammation was noted in groups in which the carrier was an OVD versus buffered saline. Fibrin accumulation was not noted in any animals in which the carrier was saline, however, all eyes with endotoxin‐spiked OVDs developed fibrin.^10^ One study that evaluated response to single intravitreal injections of various endotoxin concentrations in rabbits demonstrated similar dose‐dependent increases of aqueous flare at concentrations above 0.05 EU/eye, with signs noted as early as 4 h after injection.^11^ Vitreal cell presence was similarly dose‐related, albeit more sensitive with a no‐observable effect level of 0.01 EU/eye. Additionally, the vitreal response was delayed and had a significantly slower resolution.^11^ Evaluation of intravitreal injections of endotoxin in green monkeys found dose‐related increases in inflammation starting at 0.02 EU/eye.^12^ Inflammation required no intervention in all groups (with the highest concentration being 0.08 EU/eye), with flare, cell, and fibrin resolution within 10 days.^12^ The lower limit of detection in our study was 0.05 EU/mL, which is slightly higher than no observable effect levels reported above. While endotoxin concentrations in reported outbreaks are significantly higher than our lower limit of detection (0.5–2 EU/mL), it is possible that low levels of endotoxin are contributing to inflammation post‐operatively. If there are sub‐detectable levels of endotoxin in OVDs used during surgery, the practice of performing irrigation/aspiration at the end of phacoemulsification procedures significantly reduces viscoelastic remaining within the eye, and thus should significantly lower endotoxin levels as well. However, studies have shown that irrigation fluids are pushed into the vitreous chamber during phacoemulsification, which could act as a reservoir for endotoxin.^13^

As fibrin webs develop slightly slower compared to TASS, we proposed that perhaps slow leakage of endotoxin from the IOL may be initiated after incubation within the eye. Therefore, packaging fluid was collected for testing, then IOLs were washed, incubated, and retested; however, there was no difference in endotoxin levels after incubation. Patients could be experiencing a reaction initiated by the IOL secondary to other manufacturing residues or it may be an immune‐mediated response to the IOL material.

Limitations to this study include small sample size, limited lot numbers acquired, and an inability to detect endotoxin in the lower end of the assay's limit of detection (0.005–0.05 EU/mL). Previous validation studies have demonstrated that rFC assays perform at an equivalent or superior level compared to assays that require amebocyte lysate.^6^, ^7^ Due to the accuracy in detecting positive controls and spiked samples in our study, it is unlikely that assay sensitivity played a significant role in our findings.

As fibrin webs are frequently observed in case clusters, it is possible that endotoxin concentrations vary between batches, and lots that were evaluated in this study were not contaminated. However, OVDs from the same lots were used for phacoemulsification surgery in our hospital during the study period. IOLs from the same manufacturer but not the same lot were used as well. Two mild cases of fibrin web formation within the capsular bag and around the IOL were observed using these materials. Thus, it appears less likely that endotoxin contamination within materials used during surgery is the main cause of fibrin web formation as it occurred while using products that are known to have endotoxin less than 0.05 EU/mL. The fibrin web phenomenon is likely multifactorial and may also depend on specific patient immunogenetics or other sources of endotoxin contamination.

## 5 CONCLUSION

Evaluation using the rFC assay revealed endotoxin concentrations of less than 0.05 EU/mL in viscoelastics, intraocular lens packaging fluid, and irrigation fluid components. Incubation of IOLs prior to testing did not increase extraction of endotoxin. As fibrin webs are often observed episodically, it is possible that endotoxin levels may vary between batches, or that endotoxin does not play a main role in fibrin formation.

## CONFLICT OF INTEREST STATEMENT

None.

## Footnotes

* Endotoxin Testing Recommendations for Single‐Use Intraocular Ophthalmic Devices – Guidance for Industry and Food and Drug Administration Staff.
